# COVID-19 government measures and their impact on mental health: a cross-sectional study of older primary care patients in Germany

**DOI:** 10.3389/fpubh.2023.1141433

**Published:** 2023-05-22

**Authors:** Felix G. Wittmann, Andrea Zülke, Alexander Pabst, Melanie Luppa, Jochen René Thyrian, Anika Kästner, Wolfgang Hoffmann, Hanna Kaduszkiewicz, Juliane Döhring, Catharina Escales, Jochen Gensichen, Isabel Zöllinger, Robert Philipp Kosilek, Birgitt Wiese, Anke Oey, Hans-Helmut König, Christian Brettschneider, Thomas Frese, Steffi G. Riedel-Heller

**Affiliations:** ^1^Institute of Social Medicine, Occupational Health and Public Health (ISAP), University of Leipzig, Leipzig, Germany; ^2^Institute for Community Medicine, University Medicine Greifswald (UMG), Greifswald, Germany; ^3^German Centre for Neurodegenerative Diseases (DZNE), Site Rostock/Greifswald, Greifswald, Germany; ^4^Faculty V: School of Life Sciences, University of Siegen, Siegen, Germany; ^5^Institute of General Practice, University of Kiel, Kiel, Germany; ^6^Institute of General Practice/Family Medicine, University Hospital of LMU Munich, Munich, Germany; ^7^Hannover Medical School, Institute for General Practice, Work Group Medical Statistics and IT-Infrastructure, Hannover, Germany; ^8^Department of Health Economics and Health Service Research, University Medical Centre Hamburg-Eppendorf, Hamburg, Germany; ^9^Institute of General Practice and Family Medicine, Martin-Luther-University Halle-Wittenberg, Halle (Saale), Germany

**Keywords:** COVID-19, government measures, depression, stress, older people, social support

## Abstract

**Background:**

With the outbreak of COVID-19, government measures including social distancing and restrictions of social contacts were imposed to slow the spread of the virus. Since older adults are at increased risk of severe disease, they were particularly affected by these restrictions. These may negatively affect mental health by loneliness and social isolation, which constitute risk factors for depressiveness. We aimed to analyse the impact of perceived restriction due to government measures on depressive symptoms and investigated stress as mediator in an at-risk-population in Germany.

**Methods:**

Data were collected in April 2020 from the population of the *AgeWell.de*-study, including individuals with a Cardiovascular Risk Factors, Aging, and Incidence of Dementia (CAIDE) score ≥9, using the depression subscale of the Brief Symptom Inventory (BSI-18) and the Perceived Stress Scale (PSS-4). Feeling restricted due to COVID-19 government measures was surveyed with a standardized questionnaire. Stepwise multivariate regressions using zero-inflated negative binomial models were applied to analyse depressive symptoms, followed by a general structural equation model to assess stress as mediator. Analysis were controlled for sociodemographic factors as well as social support.

**Results:**

We analysed data from 810 older adults (mean age = 69.9, SD = 5). Feeling restricted due to COVID-19 government measures was linked to increased depressiveness (*b* = 0.19; *p* < 0.001). The association was no longer significant when adding stress and covariates (*b* = 0.04; *p* = 0.43), while stress was linked to increased depressive symptoms (*b* = 0.22; *p* < 0.001). A final model confirms the assumption that the feeling of restriction is mediated by stress (total effect: *b* = 0.26; *p* < 0.001).

**Conclusion:**

We found evidence that feeling restricted due to COVID-19 government measures is associated with higher levels of depressive symptoms in older adults at increased risk for dementia. The association is mediated by perceived stress. Furthermore, social support was significantly associated with less depressive symptoms. Thus, it is of high relevance to consider possible adverse effects of government measures related to COVID-19 on mental health of older people.

## 1. Background

With the outbreak of the SARS-CoV-2-pandemic, the World Health Organisation (WHO) has declared a global health emergency in January 2020 (1). Measures have been taken worldwide to contain the virus and as of April 2020, a third of mankind was under quarantine (2). As the government measures implement factors that potentially lead to social isolation and loneliness, they have a particular role to play for mental health. Evidence suggests that social isolation is among the strongest risk factors for depression and anxiety, especially in older people (3, 4). However, since social isolation, loneliness and depressiveness count as risk factors for dementia according to Livingston et al. (5), there is an urgent need to address the risk of these factors due to government COVID-19 measures.

After the laboratory confirmation of the first case of COVID in Germany on 28 January 2020 (6), the German government imposed the first strict restrictions on 22 March 2020 (7). These included, among others, quarantine, social and physical distancing. According to the Oxford COVID-19 Government Response Tracker of the University of Oxford, the introduction of the policy measures in Germany meant a strong, sudden restriction. On a scale from 0 to 100, the index indicates how strict measures were at a given point in time, using nine factors such as restrictions on public life or contact restrictions (8). With the regulation of 22 March 2020 in Germany, the COVID-19 stringency index rose from 32.9 to 76.7 (9).

Thus far, a review of the psychological impact of the COVID-19 pandemic on older adults outlines 18 cross-sectional surveys. While some of these studies describe less psychologically distress in older people than in younger ones, it has been demonstrated that, nevertheless, older adults experienced more severe symptoms of depression, anxiety and stress as well as loneliness during the pandemic than previous (10). Several studies have confirmed an increase in depressive symptoms comparing pre-with post-pandemic data (11). Robb et al. (12) showed an increase in reported feelings of depression symptoms (12.8% reported feeling worse), especially for women (17.3% vs. 7.8% for men). Furthermore, an association between subjective loneliness and worsened components of depression was reported. Thyrian et al. only found a small psychological impact of the pandemic for older people living at home with cognitive impairment (13). A systematic review by Giebel et al. (14) further reported an increase of depression in people living with dementia.

A systematic review by Röhr et al. (2) found evidence for psychosocial consequences of restrictions due to previous coronavirus outbreaks. Overall results of the 13 included studies were elevated risks for depression, anxiety, stress, social isolation and loneliness. Comparing people under pandemic restrictions with people who are not subject to quarantine, Liu et al. (15) founds a fivefold increased chance of depressive symptoms even 3 years after the quarantine. While a large proportion of studies from previous pandemics focuses on health care workers, most studies investigating SARS-CoV-quarantine measures suffer from little information on sociodemographic and –economic factors, for example for over 65-year-olds (2). Evidence suggests, however, that older age increases the risk for adverse mental health outcomes in times of pandemic (16). Especially isolation and physical distancing through lockdowns are affecting both mental and physical health in older adults (17). Since depression is among the most common mental health conditions in older adults (18), indication of depressiveness among older adults play a pivotal role when investigating mental health outcomes of governmental measures during pandemics.

According to Cohen et al. (19), stressors and negative life events lead to an increased risk of mental illness through perceived stress. Stress as a result of social and physical isolation due to the COVID-19 pandemic has been demonstrated several times (20–22). A high association between stress and depression regarding the COVID-19 pandemic was found by Bridgland et al. (23) analysing stressful life experiences. The possible consequences of the measures described, such as loss of social contacts, loneliness and depressiveness, are considered as risk factors for dementia (5). Since, to our knowledge, little is known about the impact of the pandemic, respectively the government measures, on older adults at risk of dementia, this paper examines the relationship between the feeling of restrictions due to COVID-19-related government measures and depressive symptoms. We further aim to examine the role of perceived stress resulting from government restrictions and depressiveness in older adults at increased risk for dementia.

## 2. Methods

### 2.1. Design and data collection

The study sample was drawn from participants of the AgeWell.de-trial (24). AgeWell.de is a multi-centred, cluster-randomized, controlled prevention trial with the primary aim of counteracting cognitive decline in older general practitioner (GP) patients at increased risk for dementia, applying a multi-component intervention (24). Participants (60–77 years; *n* = 1,030) were recruited at five study sites across Germany (Leipzig, Greifswald, Halle, Kiel and Munich) between June 2018 and October 2019. The multi-centric design has been considered a viable strategy for enlisting participants from diverse urban and rural regions. Just over half of the sample (52%) was female. A detailed description of the study sample can be found elsewhere (25). Participants had increased dementia risk according to CAIDE (Cardiovascular Risk Factors, Aging, and Incidence of Dementia; (26))—dementia risk score (CAIDE ≥9 points). The risk score is composed, among others, of vascular or metabolic risk factors, but also other pre-existing diseases. The intervention consists of advice and motivation on the modification of lifestyle factors such as optimization of diet and increased physical and social activity (25). To assess participants’ personal situation and possible impacts of the pandemic on study participation and intervention conduct, mailed paper questionnaires were sent to all participants in April 2020. At that time, the first lockdown in Germany was in force (measures were enacted on March 22nd lasting 7 weeks).

### 2.2. Measures

Questions towards the personal situation during the pandemic, like perception of personal risk or support of the government measures were surveyed using newly developed standardized questions using a 5-point Likert scale (“totally disagree” to” totally agree”). Feeling restricted due to COVID-19 government measures was surveyed by: “*I feel severely restricted by the government measures to slow the spread of the coronavirus*.”

Depressive symptoms were assessed using the German adaptation of the Brief Symptom Inventory 18 (BSI-18; (27)). The BSI-18 captures mental stress within the last 7 days. Symptoms of somatisation, depressiveness and anxiety are each asked for by six questions. For the analysis of depressive symptoms, the corresponding six items were combined into one variable after excluding cases with missing values (Cronbach’s *α* = 0.83; (27, 28)).

The Perceived-Stress Scale-4 (PSS-4), consisting of four items referring to the experience of stressful situations within the last month was chosen to identify perceived stress (29). Two questions (item 2&3) are phrased positively and have been reversed by recoding. Accordingly, a higher level of the respective sum score suggests higher perceived stress (30). The intern consistency of the four items was Cronbach’s α = 0.599. Ingram et al. (31) discuss the low internal consistency using a confirmatory factor analysis and conclude that one item (item 2) leads to a problematic structure. According to the authors, the consistency of the items was checked with and without this problematic item. The original version, including item 2, however, led to higher consistency. Therefore, the original version was used in this study. Although there is no official cut-off, Warttig et al. (30) recommends comparing it with a normative value around six points.

To control for sociodemographic variables, data collected during the baseline interview were used. Age was measured in years and calculated using the difference between date of the baseline assessment and of completion of the add-on questionnaire. Education was operationalized into three categories according to Comparative Analysis of Social mobility in Industrial Nations (CASMIN)-scale using information on vocational and professional qualifications (32). Also, all analysis were controlled for gender, allocation to control (CG) or intervention group (IG) and household size. Finally, all models were controlled for social support, assessed using the Enriched Social Support Instrument (ESSI). The instrument consists of five questions, all of which focus on perceived emotional social support (33). The five items were combined into one variable (Cronbach’s *α* = 0.88) with higher scores indicating higher social support.

### 2.3. Statistical analysis

In a first step, descriptive analysis were carried out. In addition to first results and a broad overview of the data, this provides indications of the assumption for the following multivariable analysis. Depressive symptoms were assumed to increase if a person felt restricted due to COVID-19 government measures. To analyse this association, several multivariate models were calculated.

Depressive symptoms, treated as a dependent variable for all models, shows zero inflation, i.e., a high proportion of participants reported no symptoms at all. This requires special consideration, which is why zero-inflated negative binomial regression method (ZINB) was used. ZINB treats cases that have a high level of zero differently than non-zero observations. Since no normal distribution is assumed, there is no bias caused by the zero inflation (34). Besides, over-dispersion can be resolved and ZINB has been recommended for investigations of mental health like depressive symptoms and respective influencing factors (35).

A hierarchical model was calculated to analyse the association between feeling restricted on depressive symptoms adding perceived stress in a second and covariates in a third model. Based on the assumption that stress may mediate the association between the feeling of restriction and depressiveness, a fourth model was calculated as single-mediator model to investigate stress as a mediator according to MacKinnon et al. (36). We used a generalized structural equation model for the fourth model, as it allows both the modelling of a mediator effect and zero inflation which was considered using constant dispersion.

For the correct interpretation of the ZINB models, incidence-rate ration (IRR) were calculated additionally (StataCorp 2019: 2822). Significance levels were set at the 5% level for all analysis. Besides, analysis were calculated using robust standard errors. Data management and analysis were performed using STATA, Version 16 (StataCorp, College Station, TX, United States).

## 3. Results

### 3.1. Sample characteristics

**Figure 1:**
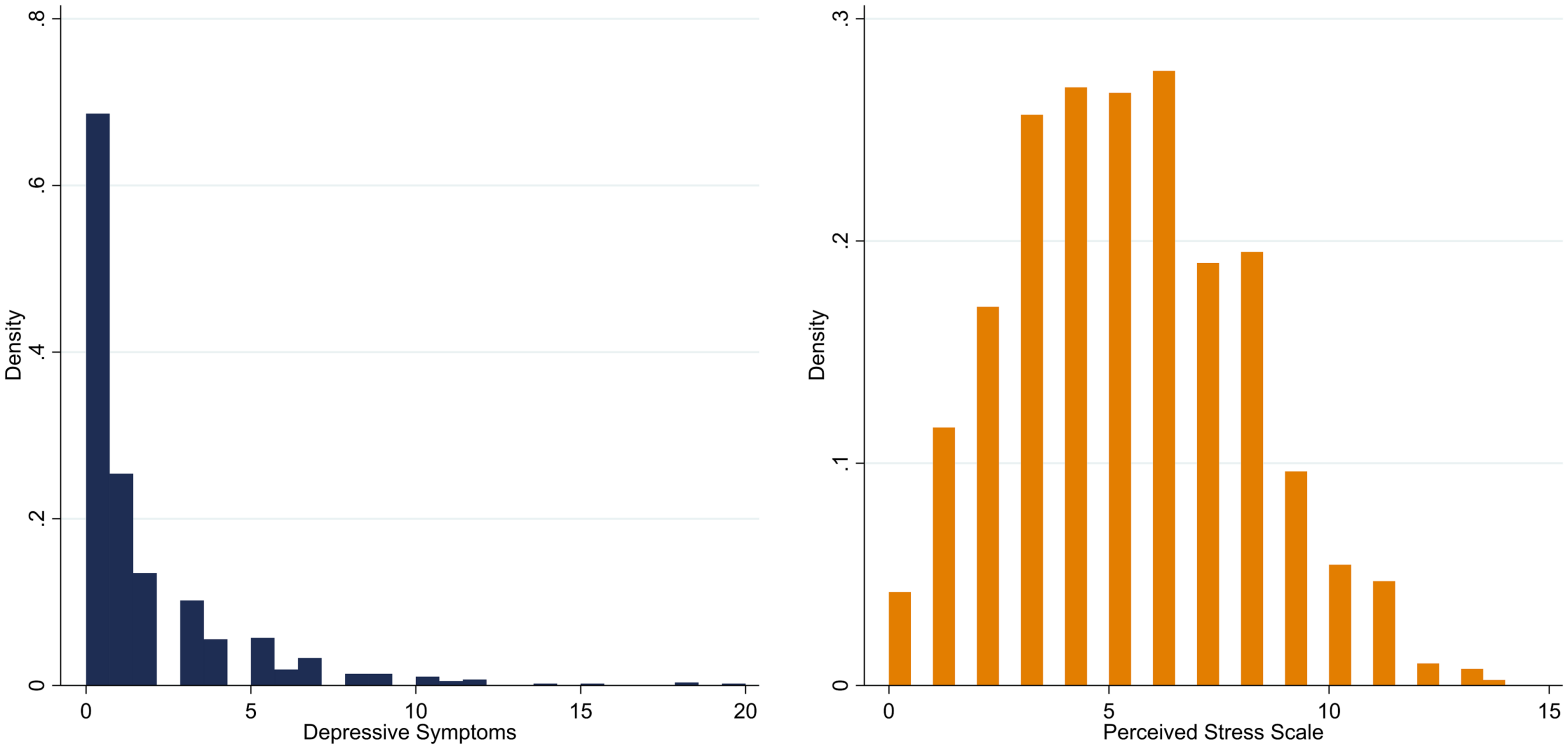
Distribution of depressive symptoms (*n* = 875) and perceived stress (*n* = 881) of the participants.

Participant characteristics are presented in Table 1. A total of 810 respondents were included for the analysis. The mean age was 69.93 (SD: 4.99) years, while 51.23% of the sample were female. 22.60% agreed or totally agreed to the statement, that they feel restricted due to the COVID-19 government measures. The majority stated that they feel partly (37.41%) restricted or rather/strongly disagreed to the statement of feeling restricted (40.00%). A high level of the value 0 was conspicuous for the severity of depressiveness (Figure 1). Within a range_low-high_ (0–20), the mean value was *x* = 1.66 (SD = 2.63). The mean value of the severity of depressiveness is 1.65 (SD: 2.64) with a high level of zero reported symptoms (49.01%, *n* = 397). Perceived stress has a mean score of 5.17 (Figure 1), whereby 69.88% have a score below the recommended reference value of six points (30).

**Table 1:** Characteristics of participants (*n* = 810).

		Missing values (*n*)	% or mean	SD	Min	Max
Age (years)		–	69.93	4.99	61	80
Gender (female)		–	51.23%	0.5	0	1
Education	Elementary	–	21.48%	0.41	0	1
	Secondary	–	52.59%	0.5	0	1
	Tertiary	–	25.93%	0.44	0	1
Feeling restricted	Strongly disagree	176	15.19%	0.36	0	1
	Rather disagree		24.81%	0.43	0	1
	Partly disagree		37.41%	0.48	0	1
	Agree		13.09%	0.34	0	1
	Totally agree		9.51%	0.29	0	1
Depressive symptoms		192	1.65	2.64	0	20
Perceived stress		186	5.17	2.66	0	14
Household size		–	1.77	0.56	1	6
Intervention group		–	45.68%	0.5	0	1
Social support		195	21.83	3.73	5	25

### 3.2. Feeling restricted due to COVID-19 measures and depressiveness

Regression results of the impact of feeling restricted due to COVID-19 government measures on depressive symptoms are shown in Table 2. With successive increase in the feeling of restriction, a significant positive increase in depressive symptoms was recorded in model 1 (*ß* = 0.194; *p* < 0.001). A high correlation was found for the inflate group, i.e., participants reporting zero depressive symptoms. The negative association indicates, that with increasing feeling of restriction, the odds of a person to report zero depressive symptoms would decrease dramatically (*ß* = −11.927; *p* < 0.001).

**Table 2:** The effect of the feeling of restriction on depressive symptoms using multiple zero-inflated-negative binomial regression models (model 1–3) and the mediating role of perceived stress using a generalized structural equation model (model 4).

	Model 1 (ZINB)		Model 2 (ZINB)		Model 3 (ZINB)		Model 4 (GSEM)	
	Coef.	95%-CI	Coef.	95%-CI	Coef.	95%-CI	Coef.	95%-CI
**Depressive symptoms**								
Feeling restricted	0.194***	(0.091; 0.297)	0.071	(−0.027; 0.170)	0.038	(−0.055; 0.131)	0.072	(−0.002; 0.147)
Perceived stress			0.223***	(0.191; 0.256)	0.221***	(0.188; 0.254)	0.222***	(0.191; 0.254)
Age (years)					0.004	(−0.015; 0.023)	0.004	(−0.012; 0.020)
Women (ref.: men)					0.034	(−0.165; 0.233)	0.036	(−0.144; 0.217)
Education (primary; ref.)					–		–	
Education (secondary)					0.175	(−0.090; 0.440)	0.048	(−0.173; 0.269)
Education (tertiary)					0.084	(−0.191; 0.359)	0.215	(−0.014; 0.443)
Household size					−0.087	(−0.282; 0.108)	−0.219***	(−0.394; −0.044)
Social support					−0.035***	(−0.058; −0.013)	−0.062***	(−0.080; −0.045)
Intervention Group					0.217***	(0.024; 0.411)	−0.031	(−0.200; 0.139)
**Inflate (zero depressive symptoms)**								
Feeling restricted	−11.927***	(−20.681; −3.174)	−0.129	(−0.409; 0.152)	−0.333	(−0.695; 0.029)	–	
Perceived stress			−0.133***	(−0.223; −0.044)	−0.057	(−0.177; 0.063)	–	
Age (years)					0.023	(−0.044; 0.090)	–	
Women (ref.: men)					−0.216	(−0.840; 0.409)	–	
Education (primary; ref.)					–		–	
Education (secondary)					0.549	(−0.556; 1.654)	–	
Education (tertiary)					−0.325	(−1.438; 0.787)	–	
Household size					0.607	(−0.054; 1.268)	–	
Social support					0.329***	(0.090; 0.567)	–	
Intervention Group					1.040***	(0.130; 1.951)	–	
**Perceived stress**								
Feeling restricted							0.394***	(0.229; 0.559)
**Total mediation effect**							0.295***	(0.223; 0.366)
Obs.	810		810		810		810	
Nonzero obs.	413		413		413		413	
AIC	2755.753		2559.243		2505.604		6393.373	

CI, Confidence Interval; AIC, Akaike Information criterion; GSEM, Generalized structural equation model; ZINB, Zero-inflated negative binomial regression; Men (Gender) and Primary education as reference categories; Robust standard errors; Significance level: *p < 0.05, **p < 0.01, ***p < 0.001.

When adding perceived stress, the association between feeling of restriction and depressive symptoms disappears, while there is a significant correlation between perceived stress and depressiveness (model 2; *ß* = 0.223; *p* < 0.001). For the group of participants with zero reported depressive symptoms, there is a negative, significant association with perceived stress (*ß* = −0.133; *p* < 0.001). Together, these results show that with increasing perceived stress, the odds of a person to report depressive symptoms increases by an IRR of 1.25, while the odds to report zero depressive symptoms decrease by an IRR of 0.88.

Finally, covariates were added to control for relevant factors in model 3. As in model 2, perceived stress was positively associated with depressive symptoms (*ß* = 0.221; *p* < 0.001), while no association was detected with feeling restricted. Perceived stress was no longer significant in the inflate group, i.e., participants reporting zero depressive symptoms. Furthermore, social support was negatively associated with depressive symptoms (*ß* = −0.035; *p* < 0.001) and positively associated with reporting zero depressive symptoms (*ß* = 0.329; *p* < 0.001). A positive association between the allocation to the intervention group and depressive symptoms was observed (*ß* = 0.217; *p* < 0.001), while there was also a positive association between intervention group allocation and reporting zero depressive symptoms.

A closer look at the Akaike information criterion (37) shows that model 3 fits the data best compared to the preceding models.

### 3.3. The mediating role of stress

Our study assumed a mediating effect of stress on the relation between feelings of restriction and depressive symptoms. The results indicate a full mediation of the hypothesized effect (Table 2; model 4). We observed a positive, significant association between perceived stress and depressive symptoms (*b* = 0.222; *p* < 0.001), while there is no association between feelings of restriction and depressive symptoms (*b* = 0.072; *p* = 0.06). Furthermore, an association between the feeling of restriction and perceived stress was observed (*ß* = 0.394; *p* < 0.001). The total effect of feeling restricted due to COVID-19 government measures and perceived stress on depressive symptoms is *ß* = 0.295 (*p* < 0.001). Moreover, there were significant correlations between household size and depressive symptoms (*ß* = −0.219; *p* < 0.001) and social support and depressive symptoms (*ß* = −0.062; *p* < 0.001).

Since the mediating effect of stress can be measured only in participants reporting a non-zero level of depressive symptoms, no results of the GSEM are reported for the inflate group.

## 4. Discussion

In the current study, we aimed to analyse the association between the feelings of restriction due to COVID-19 government measures and depressive symptoms in a sample of older adults at increased risk for dementia in Germany. We further investigated the effect of stress and, lastly, the role of stress as mediator between feelings of restriction due to COVID-19 government measures and depressive symptoms.

In our study, about a half of the participants reported some level of depressive symptoms. Other studies found somewhat lower levels of depressiveness. Petrowski et al. report a mean of the depression subscale of 1.56(SD = 2.9) for people between 65 and 69 years prior to the pandemic situation (38). Gerhards et al. (39) report a mean value of 1.36(SD = 0.44) in a representative sample of older people from Germany, whereby the data were collected during the first lockdown, covering the same timespan as our data. An increase during the outbreak of COVID-19 has been reported in many studies, Bueno-Notivol et al. (40) report even a seven-time higher prevalence of depressiveness (3.44% vs. 25%) in a meta-analysis including 12 studies compared with prevalence of depressiveness in 2017.

We found a significant association between the feelings of restriction due to COVID-19 government measures and depressive symptoms. The association persists even when controlling for covariates (sociodemographic characteristics, household size, social support and belonging to intervention group; results not shown). These results are in line with other investigations analysing depressiveness during COVID-19 (10, 11, 40–44). While most studies analysing depressiveness during pandemic situations solely focus on impacts of the pandemic itself, to our knowledge, only few investigations concentrated on the government measures and their respective impact on mental health (45, 46). As outlined above, focussing on pandemic-related restrictions is important due to the aspect of involuntary social isolation (47). Further, it is important to take a closer look on subjective feelings linked to the respective measures since they are of particular importance for the relationship between social isolation and mental health (48). The results of this study underline the importance of the risk of depressiveness due to the COVID-19 restrictions, especially against the background that, according to Livingston et al. (5), depressiveness constitutes a risk factor for dementia.

In several multi-variable regression models, we found that feelings of restriction due to COVID-19 government measures are associated with increased depressiveness, but only if perceived stress is not accounted for. Perceived stress itself was independently associated with higher levels of depressive symptoms, rendering the positive association with feelings of restriction insignificant. This is in agreement with overwhelming evidence that stress is closely associated with depressive symptoms (49–52), especially during pandemic periods (53–56). Nevertheless, most of the studies relating stress and depression during pandemic situations refer to populations like students or health care workers. Our results add to the evidence that a strong association of perceived stress related to COVID-induced restrictions with depression poses a substantial health burden especially in older people.

We further found that the association of feeling restricted by governmental measures and depressive symptoms was fully mediated by perceived stress. This result is in line with another study reporting stress due to restrictions (45). A possible explanation for the association between the feeling of restricted and stress might be a strong correlation between stress and loneliness in our data that is in line with other studies (57, 58).

Social support was negatively associated with depressive symptoms in our study, and positively associated with the absence of depressive symptoms, respectively. Although the result is in line with previous findings (59–61), it is of interest to the context of pandemic situation and government restrictions. As Liu et al. (62) reports, social support can mediate the relation between loneliness and depressive symptoms in older adults, which highlights the importance of social support. In exceptional situations, such as in pandemic periods, the social environment is considered to be a protective factor for mental health (63, 64). Accordingly, as an indicator of social environment, household size was negatively associated with depressive symptoms in our analysis (model 4).

Belonging to the intervention group of the AgeWell.de-trial was associated with higher levels of depressive symptoms. Since baseline interviews were conducted between June 2018 and October 2019, intervention duration varied considerably between participants at the time of data collection (April 2020). Accordingly, the observed association of group allocation and depressive symptoms cannot be interpreted as a causal intervention effect at the given point in time. Analysis of intervention effects on depressive symptomatology at follow-up are currently pending. However, a significant difference was found between participants who dropped out between the baseline interview and distribution of the add-on questionnaire. The mean value of depressive symptoms was significantly higher in control group-participants who dropped out of the study than among intervention group dropouts (mean IG: *n* = 32, *ß* = 1.21 vs. CG: *n* = 32; *ß* = 2.34; *p* = 0.034; see Supplementary Figure S1).

## 5. Limitation and strengths of the study

Being limited to cross-sectional data, this study cannot make conclusions about changes in depressive symptoms from pre-pandemic due to the restrictions. It is therefore not possible to make causal interpretations regarding the association of governmental restrictions to contain the spread of COVID-19 and depressive symptoms. In addition, it must be stressed that at the time of the data collection, the government measures had only been in effect for a few weeks. Based on the results of this study, it would be relevant to examine the extent to which stress and depressiveness and their respective association may have increased further with time. Persistent states of unusual situations or negative life events are particularly relevant to the relationship between stress and depression, since the length and severity of circumstances matters (49). Furthermore, the sensitivity analysis revealed that participants with higher levels of baseline depressiveness were less likely to complete the add-on questionnaire for the COVID survey (*p* = 0.026).

Since the sample of this investigation is characterised by increased risk of dementia due to the CAIDE-score, our sample may demonstrate a higher prevalence of pre-existing disease. With a generalization to the general population is therefore only possible to a limited extent. Notwithstanding these limitations, the study suggests results for older primary care patients as a relevant group.

Finally, it must be stressed that this study focused on depressive symptoms. Investigating depressive symptoms in older adults is of particular importance due to the high prevalence and comorbidity. Nevertheless, an issue that was not addressed in this study was the effect of restrictions due to COVID-19 government measures on other mental health outcomes. It would be of interest, to further investigate anxiety, somatisation or other mental health outcomes as an outcome of pandemic restrictions.

## 6. Conclusion

This study set out to analyse the effect of feeling restricted due to government measures to slow the spread of COVID-19 on depressive symptoms in a sample of older adults at risk for dementia in Germany. The second aim of this study was to further investigate the effect of stress on the respective association. Our findings support the need of a general discussion of effects of government measures during pandemics on mental health of older adults. Restrictions protect the health of people and prevent deaths, but they also harbours risk of loneliness, lack of social support, increasing stress and finally higher depressive symptoms. With regard to comorbidity with other relevant diseases, such as dementia, these risks are of particular importance for older adults. The relevance of social support is clearly supported by the current findings, whereby adequate risk communication and mental health recommendations could be reasonable approaches to tackle feelings of restriction and perceived stress and thereby reduce the risk for depressive symptoms.

## Data availability statement

**The datasets are not publically available due to privacy restrictions:** Individual participant data underlying the results of this article, after de-identification, is available to researchers who submit a methodologically sound proposal to the AgeWell.de steering commitee (correspondence: stiffi.riedle-heller@medizin.uni-leipzig.de) for use of data in the approved proposal. Requests to access the datasets should be directed to SR-H, steffi.riedel-heller@medizin.uni-leipzig.de.

## Ethics statement

The studies involving human participants were reviewed and approved by Ethical Committee at the Medical Faculty, Leipzig University; Ethical Committee at the Medical Faculty, Christian-Albrechts-University, Kiel; Ethical Committee at Universitätsmedizin Greifswald; Ethical Committee at the Medical Faculty, Ludwig-Maximilian-University, Munich; Ethical Committee at the Medical Faculty, Martin-Luther-University Halle-Wittenberg; Ruprecht-Karls-University, Heidelberg. The patients/participants provided their written informed consent to participate in this study.

## Author contributions

JG, WH, HK, H-HK, JT, BW, and SR-H: conceptualization of the *AgeWell.de*-trial. FW: analysis and interpretation of data and writing the original draft. JG, WH, HK, H-HK, JT, BW, and SR-H: funding acquisition. ML, AZ, AP, TF, JG, WH, HK, H-HK, JT, BW, and SR-H: supervision. ML, AZ, AP, JT, AK, WH, HK, JD, CE, JG, IZ, RK, BW, AO, H-HK, CB, TF, and SR-H: review and editing. All authors contributed to the article and approved the submitted version.

## Funding

Open Access funding enabled and organized by the Open Access Publishing Fund of Leipzig University supported by the German Research Foundation within the program Open Access Publication Funding. This publication is part of the study “*AgeWell.de – a multi-centric cluster-randomized controlled prevention trial in primary care*” and was funded by the German Federal Ministry for Education and Research (*BMBF*; grants: *01GL1704A, 01GL1704B, 01GL1704C, 01GL1704D, 01GL1704E,* and *01GL1704F)*.

## Conflict of interest

The authors declare that the research was conducted in the absence of any commercial or financial relationships that could be construed as a potential conflict of interest.

## Publisher’s note

All claims expressed in this article are solely those of the authors and do not necessarily represent those of their affiliated organizations, or those of the publisher, the editors and the reviewers. Any product that may be evaluated in this article, or claim that may be made by its manufacturer, is not guaranteed or endorsed by the publisher.
