# Supplementary material for: COVID-19 government measures and their impact on mental health: a cross-sectional study of older primary care patients in Germany
Source: Front Public Health. 2023 May 22;11:1141433. doi: 10.3389/fpubh.2023.1141433 (PMC10239963; doi:10.3389/fpubh.2023.1141433)
Supplement: Supplementary file 1 [file Table_1.DOCX]

Supplementary Material

Covid-19 government measures and their impact on mental health: a cross-sectional study of older primary care patients in Germany

**Felix G. Wittmann^1*^, Andrea Zülke^1^, Alexander Pabst^1^, Melanie Luppa^1^, Jochen René Thyrian^2,3,4^, Anika Kästner^2^, Wolfgang Hoffmann^2,3^, Hanna Kaduszkiewicz^5^, Juliane Döhring^5^, Catharina Escales^5^, Jochen Gensichen^6^, Isabel Zöllinger^6^, Robert Philipp Kosilek^6^, Birgitt Wiese^7^, Anke Oey^7^, Hans-Helmut König^8^, Christian Brettschneider^8^, Thomas Frese^9†^, Steffi G. Riedel-Heller^1†^**

**^†^ Shared last authorship**

*** Correspondence:** Corresponding Author: [Felix.Wittmann@medizin.uni-leipzig.de](mailto:Felix.Wittmann@medizin.uni-leipzig.de)

**Supplementary Figure 1.** Mean of Depressive Symptoms for Dropouts stratified by allocation


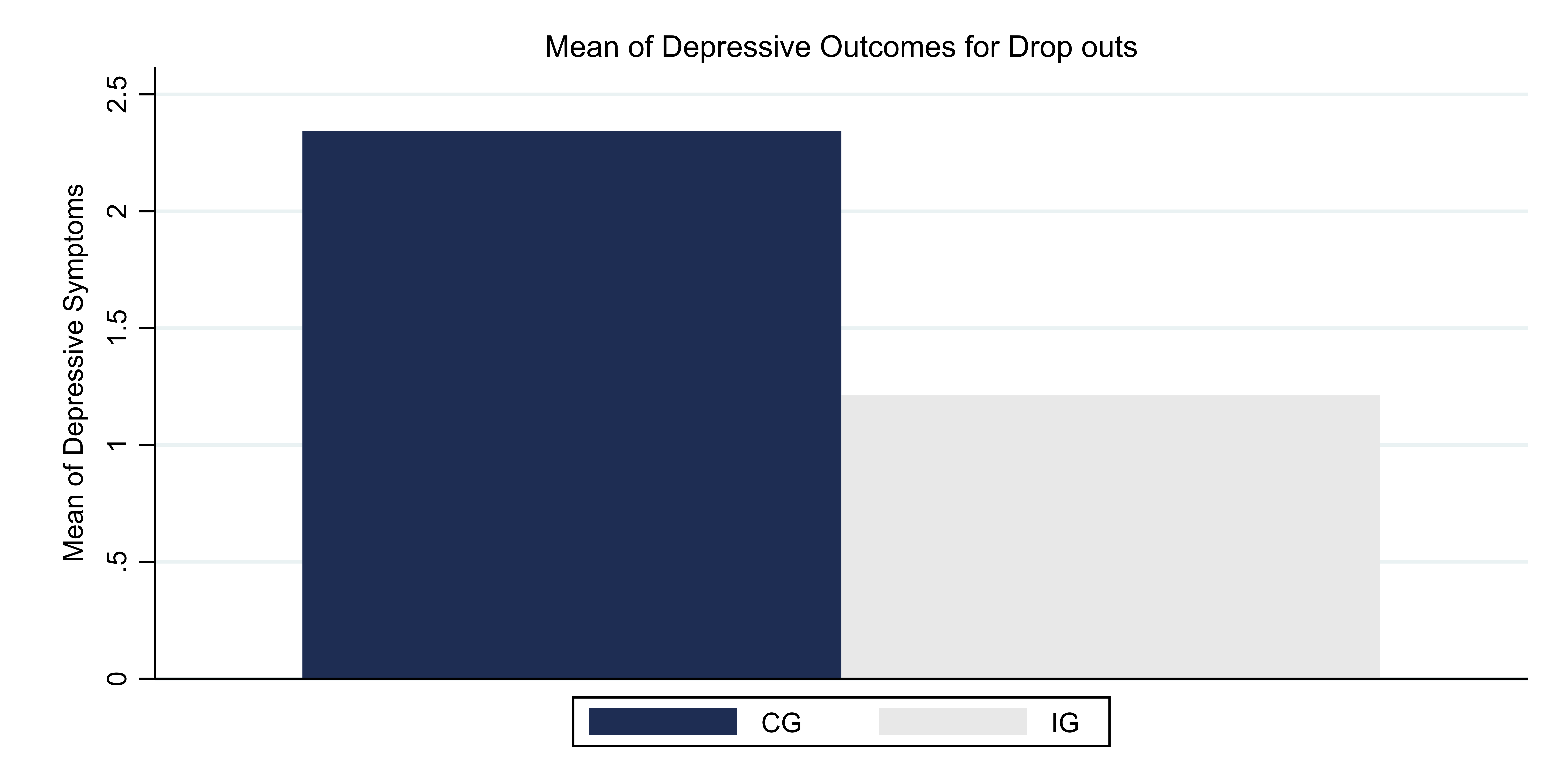


**Supplementary Figure 1.** CG: control group; IG: intervention group
